# Enhanced optoelectronic performances of vertically aligned hexagonal boron nitride nanowalls-nanocrystalline diamond heterostructures

**DOI:** 10.1038/srep29444

**Published:** 2016-07-11

**Authors:** Kamatchi Jothiramalingam Sankaran, Duc Quang Hoang, Srinivasu Kunuku, Svetlana Korneychuk, Stuart Turner, Paulius Pobedinskas, Sien Drijkoningen, Marlies K. Van Bael, Jan D’ Haen, Johan Verbeeck, Keh-Chyang Leou, I-Nan Lin, Ken Haenen

**Affiliations:** 1 Institute for Materials Research (IMO), Hasselt University, 3590 Diepenbeek, Belgium; 2 IMOMEC, IMEC vzw, 3590 Diepenbeek, Belgium; 3 Department of Engineering and System Science, National Tsing Hua University, 30013 Hsinchu, Taiwan; 4 Electron Microscopy for Materials Science (EMAT), University of Antwerp, 2020 Antwerp, Belgium; 5 Department of Physics, Tamkang University, 251 Tamsui, Taiwan

## Abstract

Field electron emission (FEE) properties of vertically aligned hexagonal boron nitride nanowalls (hBNNWs) grown on Si have been markedly enhanced through the use of nitrogen doped nanocrystalline diamond (nNCD) films as an interlayer. The FEE properties of hBNNWs-nNCD heterostructures show a low turn-on field of 15.2 V/μm, a high FEE current density of 1.48 mA/cm^2^ and life-time up to a period of 248 min. These values are far superior to those for hBNNWs grown on Si substrates without the nNCD interlayer, which have a turn-on field of 46.6 V/μm with 0.21 mA/cm^2^ FEE current density and life-time of 27 min. Cross-sectional TEM investigation reveals that the utilization of the diamond interlayer circumvented the formation of amorphous boron nitride prior to the growth of hexagonal boron nitride. Moreover, incorporation of carbon in hBNNWs improves the conductivity of hBNNWs. Such a unique combination of materials results in efficient electron transport crossing nNCD-to-hBNNWs interface and inside the hBNNWs that results in enhanced field emission of electrons. The prospective application of these materials is manifested by plasma illumination measurements with lower threshold voltage (370 V) and longer life-time, authorizing the role of hBNNWs-nNCD heterostructures in the enhancement of electron emission.

Field emission electron sources are the vital building blocks in an array of appliances, including microwave amplifiers, e-beam induced light sources, flat panel displays and travelling wave tubes^1^,^2^,^3^,^4^,^5^,^6^. In the last few years, the design, realization and application of a new generation of cold cathodes based on advanced nanomaterials have been the object of remarkable interest by researchers. The intention has been to find innovative nanomaterials possessing the best possible FEE performances in terms of low turn-on field, high FEE current density and robustness. Recent consideration has been paid to diamond^7^, aluminum nitride (AlN)^8^, and boron nitride (BN)^9^,^10^, with negative electron affinity (NEA), and carbon nanotubes^11^,^12^ with a large field enhancement factor.

For a high-quality electron field emitter, adequate electrons supply from the substrates (the Si) to the emitting sites (the hBNNWs) is critical, besides the low work function for the emitting surface. Combination of two chemically different nanostructured materials in a heterostructure is a versatile building method for modern nanodevices. Compared to single component structures, heterostructures habitually reveal enhanced characteristics, such as emission efficiency^13^ and high electron mobility^14^, which are the significant factors for many device performances^15^,^16^,^17^. A myriad of heterostructured materials have been developed. Heterostructures such as single-walled CNTs–CdSe quantum dots^18^, SnO_2_-CNTs^19^, MoS_2_–WS_2_^20^, CNT-graphene^21^, gold–ultrananocrystalline diamond (UNCD)^22^, BN–graphene^23^, CNT–UNCD on Si tips^24^, ZnO nanopillars–Si^25^, ZnO nanorods–UNCD^26^, graphene-nanodiamond^27^, InAs–GaAs nanowires^28^, TiSi_2_ nanonets–Si nanostructures^29^ have been extensively studied. Fabrication of such heterostructures is not only vital for fundamental studies but also for diverse advanced functional devices, for instance, interconnects and emitters, etc.

Being encouraged by a distinctive possibility to merge two nanostructured materials, here we set out to investigate novel vertically aligned hexagonal boron nitride nanowalls (hBNNWs)-nanocrystalline diamond (NCD) heterostructures and to examine their FEE and plasma illumination (PI) properties. The bonding structure and microstructure of hBNNWs-NCD heterostructures were detailed characterized to better understand the possible mechanism of the growth of hBNNWs on NCD films and the enhanced FEE properties of these heterostructures.

## Results and Discussion

**Figure 1:**
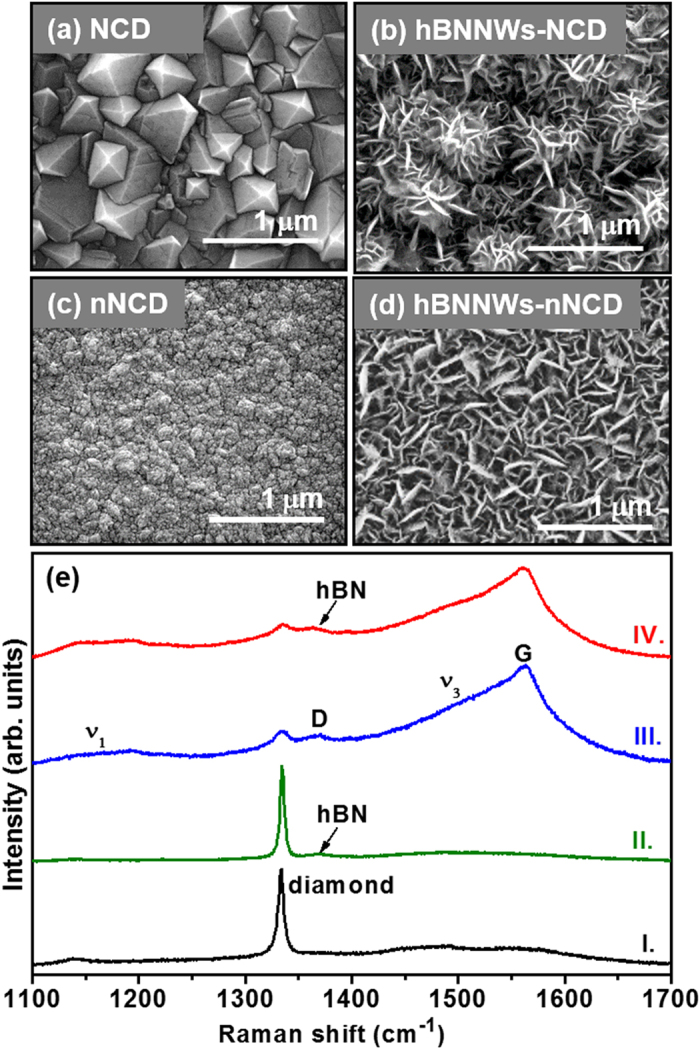
The plane view SEM micrographs of (**a**) NCD films, (**b**) hBNNWs-NCD, (**c**) nNCD films and (**d**) hBNNWs-nNCD. (**e**) micro-Raman spectra for I. NCD films, II. hBNNWs-NCD, III. nNCD films and IV. hBNNWs-nNCD.

The fabrication of hBNNWs-NCD heterostructures was a two-step process. In the first step, two types of NCD films (undoped NCD and nitrogen doped NCD (nNCD)) were grown on mirror polished (100)-oriented silicon (Si) wafers using an ASTeX 6500 series microwave plasma enhanced chemical vapor deposition (MPECVD) reactor. Figure 1a shows an SEM image of an undoped NCD film grown on the Si substrate. The entire surface was very rough and uniformly covered with randomly oriented diamond grains with clearly distinguishable edges and facets and having grain sizes of 0.3–0.8 μm. The SEM image of nNCD films shown in Fig. 1c reveals a dramatic change in the surface morphology of the films when adding N_2_ in the CH_4_/H_2_ microwave plasma. The sharp-edged crystal structure is replaced by a roundish granular structure with nano-sized grains. In the second step, hBNNWs were grown on the NCD films by a home-built unbalanced 13.56 MHz radio frequency (RF) sputtering technique^30^,^31^. Figure 1b,d show the surface morphology of hBNNWs grown on the diamond films. Both NCD film surfaces were uniformly covered with a layer of two dimensional nanostructures (nanowalls) with a compact and curled morphology. It is to be noted that the SEM morphology of hBNNWs is closely related with granular structure of the underneath NCD films. For the NCD films, the hBNNWs formed micron-sized aggregates, which seem to grow on top of each diamond grain of the NCD films separately (Fig. 1b), whereas, for nNCD films, the hBNNWs formed a more uniform layer that is closely related to the very smooth surfaces of the nNCD films (Fig. 1d). The hBNNWs were vertically aligned with respect to the plane of the NCD substrates. They were mutually interconnected forming an extended network. In contrast, a non-uniform and unbranched growth of hBNNWs was observed when the nanowalls were grown on Si substrates (Fig. S1, Supporting Information).

Further analysis of these samples was carried out by micro-Raman spectroscopy employing a laser beam with wavelength of 488 nm and spot size of ~1 μm. Spectrum I in Fig. 1e acquired from NCD film on Si shows a peak at 1334 cm^−1^, the characteristic F_2g_ band for diamond lattices. Small broad resonance peaks near 1138 cm^−1^, 1480 cm^−1^ and 1554 cm^−1^ are also present; these peaks are associated with disordered carbon^32^,^33^. The Raman spectrum of these films was markedly modified when N_2_ species were incorporated into the plasma in the growth of nNCD films. The Raman spectrum of the nNCD films (spectrum III, Fig. 1e) contains *ν*_1_-band (1185 cm^−1^) and *ν*_3_-band (1532 cm^−1^) resonance peaks, which correspond to the deformation modes of CH_x_ bonds in the nNCD films^34^, and D-band (1368 cm^−1^) and G-band (1562 cm^−1^) resonance peaks, which correspond to *sp*^2^ bonded carbon, i.e., the disordered carbon and graphite^35^,^36^,^37^,^38^. The diamond (1334 cm^−1^) resonance peak, which corresponds to the F_2g_ resonance mode of the 3C diamond lattice, is only barely visible. The invisibility of the diamond resonance peak does not mean that the films comprise no *sp*^3^-bonded carbon. It is due to the fact that the Raman signal is overwhelmingly more sensitive to the *sp*^2^-bonded carbon than to the *sp*^3^-bonded ones that renders the diamond peak remains relatively feeble^37^. On the other hand, hBNNWs-Si materials contain a major peak that appears at 1368 cm^−1^, attributed to the high frequency intralayer E_2g_ vibration mode of hBN (spectrum I of Fig. S2, Supporting Information)^39^,^40^,^41^,^42^. Since the hexagonal boron nitride (hBN) material is porous in nature, only a small peak corresponding to the hBN signal (indicated by an arrow in spectra II and IV) is observed at 1366 cm^−1^ for hBNNWs-NCD and hBNNWs-nNCD samples. The hBN peak is overlapped with the D band (1368 cm^−1^) of the diamond films (spectra II and IV, Fig. 1e).

**Figure 2:**
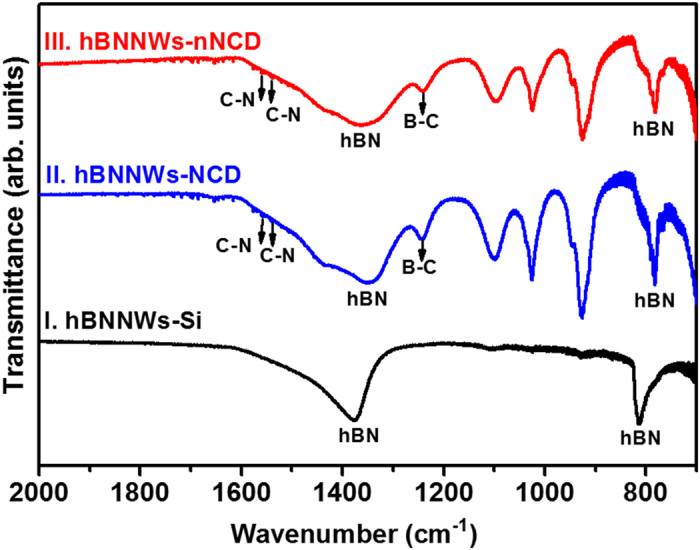
FTIR spectra for I. hBNNWs-Si, II. hBNNWs-NCD and III. hBNNWs-nNCD. FTIR transmission spectra were undertaken vertically to the film surface in the range frequency of 400–4000 cm^−1^ with a resolution of 2.0 cm^−1^.

FTIR spectroscopy measurements were performed to further investigate the bonding characteristics of these heterostructures. Spectrum I of Fig. 2 for hBNNWs-Si shows one sharp absorption peak at 812 cm^−1^ and one broad absorption band with the bottom in the range of 1300–1500 cm^−1^, which was attributed to the A_2u_ (B-N-B bending vibration mode parallel to the *c*-axis) and E_1u_ (B-N stretching vibration mode perpendicular to the *c*-axis) modes of hBN^43^,^44^,^45^, respectively, validating their good crystallinity. Spectra II and III of Fig. 2 for hBNNWs-NCD and hBNNWs-nNCD heterostructures are very alike. Both spectra illustrate the characteristic peaks of hBN. In addition, the peak at 1238 cm^−1^ can be consigned to the stretching vibration of boron-rich (or carbon-rich) B-C bonds^46^,^47^. These observations imply that there are some carbon species incorporated into the hBNNWs films. The absorption band centered at 1238 cm^−1^ may also be associated to the stretching vibration of C-N bonds^47^,^48^. Furthermore, the formation of *sp*^2^ C-N bonds could contribute to the small absorption peaks at ~1542 cm^−1^ and 1564 cm^−1^, respectively^46^,^49^. Notably, the stretching vibration of C-N bonds are expected to give an absorption band centered at 1238 cm^−1^, but it cannot be clearly resolved from the B-C bands.

**Figure 3:**
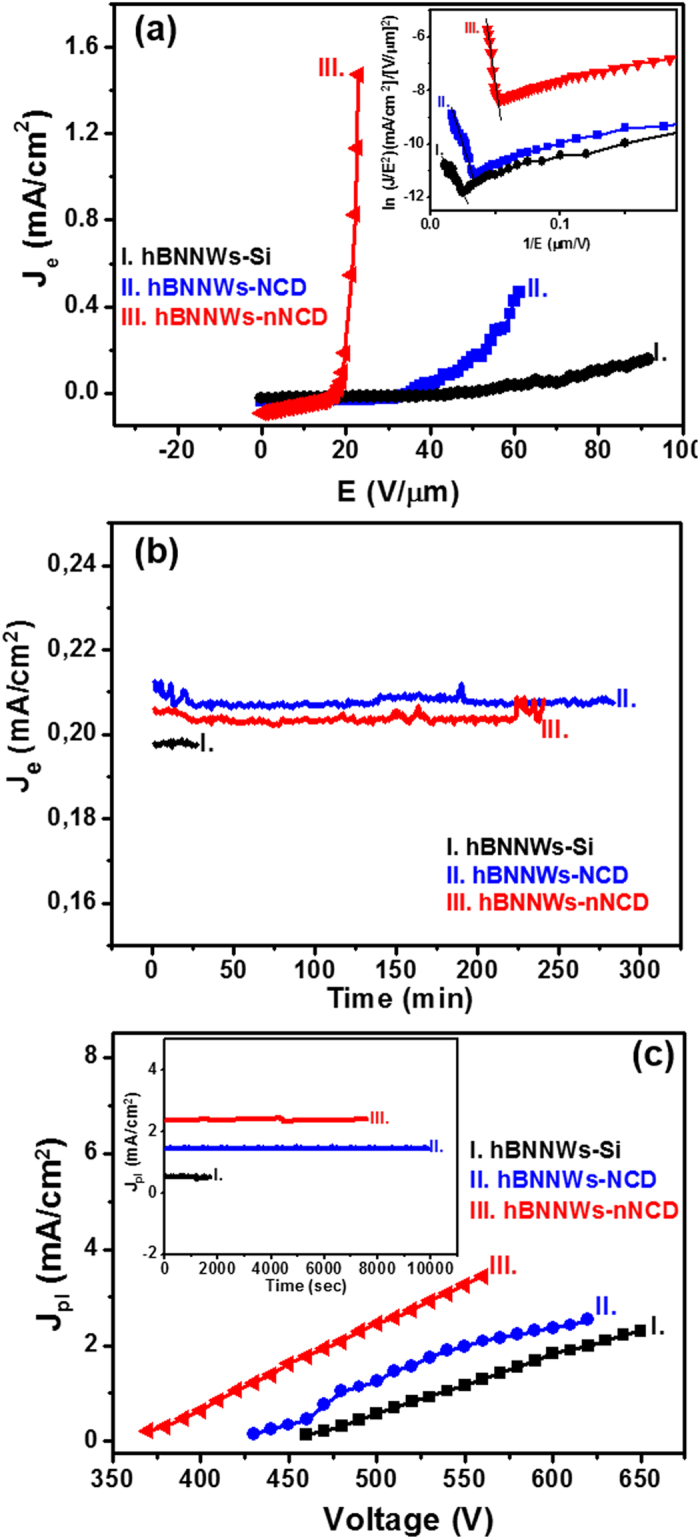
(**a**) The field electron emission properties (*J*_e_-*E* curves) and (**b**) the current density versus time curves of I. hBNNWs-Si, II. hBNNWs-NCD, III. hBNNWs-nNCD. The inset in “a” shows the corresponding Fowler-Nordheim plots. (**c**) The plasma current density versus applied voltage with the inset showing the plasma illumination stability, the life-time, of the microplasma cavities, which were fabricated using I. hBNNWs-Si, II. hBNNWs-NCD and III. hBNNWs-nNCD as cathode materials.

The FEE properties of hBNNWs-diamond heterostructures are derived through the FEE current density (*J*_e_)−applied field (*E*) curves, which are illustrated in Fig. 3a. The Fowler-Nordheim (F-N) theory could very well describe the FEE phenomenon^50^. The corresponding F-N curves are plotted as insets. The *E*_0_ value for inducing FEE process decreased from 46.6 V/μm for hBNNWs-Si (curve I, Fig. 3a) to 35.5 V/μm for hBNNWs-NCD (curve II, Fig. 3a), whereas the *J*_e_ value increased from 0.21 mA/cm^2^ at an applied field of 91.6 V/μm for hBNNWs-Si to a value of 0.46 mA/cm^2^ at an applied field of 61.3 V/μm for hBNNWs-NCD. The FEE properties are even better for the hBNNWs-nNCD, which used nNCD films as interlayer, viz., with the smallest *E*_0_ value of 15.2 V/μm and the highest *J*_e_ value of 1.48 mA/cm^2^ (at an applied field of 21.3 V/μm, curve III, Fig. 3a). Furthermore, a plot of log (*J*_e_/*E*^2^) versus (1/*E*), the so-called F-N plot inset of Fig. 3a, gives a straight line. The literal field enhancement factor (*β*) value can be calculated from the F-N ‘slope’ using the relation: *β* = [−6.8 × 10^3^ *φ*^3/2^]/*m,* where *m* is the slope of the F-N plot. From the slopes of the F-N plots the *β* values of these heterostructures were calculated by taking the *φ* value of hBN as 6.0 eV^51^. The hBNNWs-Si exhibits the lowest *β* value of 560. The *β* value increases to 2110 for hBNNWs-NCD, which is almost 4 times as much that of the hBNNWs-Si. Upon nitrogen doping, the *β* value further increases to 3057. These FEE parameters are listed in Table S1.

The stability of the FEE current is an imperative parameter associated to the applications of cold cathode materials. The life-time stability measurements for these films were evaluated by measuring the *J*_e_ versus time curves of these heterostructures. Fig. 3b shows that, ignoring short-term fluctuations owing to adsorption and desorption of residual gas molecules and diffusion of adsorbed species on the emitter surface, the emission current variations corresponding to *J*_e_ of 0.21 mA/cm^2^ recorded over a period of 284 min for hBNNWs-NCD at a working field of 53.5 V/μm and 248 min for hBNNWs-nNCD corresponding to *J*_e_ of 0.20 mA/cm^2^ at a working field of 19.6 V/μm before the start of degradation (curves II and III, Fig. 3b). In contrast, the hBNNWs-Si (curve I, Fig. 3b) shows the emission current variations recorded only a period of 27 min at a working field of 90.0 V/μm corresponding to *J*_e_ of 0.198 mA/cm^2^. It is worth noting that even though the hBNNWs-nNCD films show slightly inferior life-time stability to the hBNNWs-NCD films, the robustness of these films is still much better than the life-time stability of conventional EFE materials. The comparison on the FEE parameters of hBNNWs-nNCD with other field emitting materials^52^,^53^,^54^,^55^,^56^,^57^,^58^,^59^ reported in literature, is summarized in Table 1.

The challenging developments in the hardiness of the hBNNWs-NCD heterostructures can be better exemplified by using these field electron emitters as cathodes for a microplasma device, as the cathodes are subjected to energetic Ar-ions bombardment in this device, which is considered as the harshest environment for electron emitters^60^,^61^. Figure S3 in Supporting Information shows that the microplasma devices using hBNNWs-NCD heterostructures as cathode exhibit a superior plasma illumination (PI) behavior to those using hBNNWs-Si as cathodes. The microplasma devices using the hBNNWs-nNCD as cathode can be triggered by a voltage as low as 370 V (image series III, Fig. S3 in the Supporting Information), whereas those using hBNNWs-NCD as cathode need 430 V to ignite the plasma (image series II, Fig. S3 in the Supporting Information). In contrast, the hBNNWs-Si based microplasma devices need a higher voltage, around 460 V (image series I, Fig. S3 in the Supporting Information), to trigger the plasma. The plasma intensity enhances monotonically with the applied voltage. The superior PI characteristics are better illustrated by the variation of the plasma current density (*J*_pl_) versus the applied voltage. Figure 3c indicates that the *J*_pl_ values increased monotonously with the applied voltage and reached 1.27 mA/cm^2^ and 2.46 mA/cm^2^, at an applied voltage of 500 V, for hBNNWs-NCD and hBNNWs-nNCD based microplasma devices (curves II and III, Fig. 3c), respectively, whereas only a *J*_pl_ = 0.57 mA/cm^2^ at an applied voltage of 500 V was achieved for the hBNNWs-Si based ones (curve I, Fig. 3c).

To estimate the constancy of the PI performance from hBNNWs-diamond based microplasma devices, the plasma current was observed over a long period with a constant applied voltage of 500 V. It should be mentioned that under this applied voltage, the *J*_pl_-value is 1.45 mA/cm^2^ for hBNNWs-NCD and is 2.35 mA/cm^2^ for hBNNWs-nNCD based microplasma devices. Under this test condition, the PI intensity of the hBNNWs-NCD and hBNNWs-nNCD based microplasma devices continues to be stable over 163 min and 122 min, respectively, displaying the high stability of the hBNNWs-diamond based microplasma devices (curves II and III, inset of Fig. 3c). That the nNCD films is less stable than the NCD films can be ascribed to the presence of nanographitic phase in the former materials that, in turn, resulted in shorter life-time for hBNNWs-nNCD heterostructures than that of hBNNWs-NCD heterostructures. In contrast, the *J*_pl_ value of 0.54 mA/cm^2^ (at constantly applied 500 V) decreased fast after 28 min of plasma ignition for the hBNNWs-Si-based microplasma devices (curve I, inset of Fig. 3c). Consequently, the better PI performance of the microplasma devices based on the hBNNWs-diamond heterostructures, as associated with that of hBNNWs-Si based ones, is closely interrelated with the enhanced FEE properties of the hBNNWs-diamond heterostructures.

**Figure 4:**
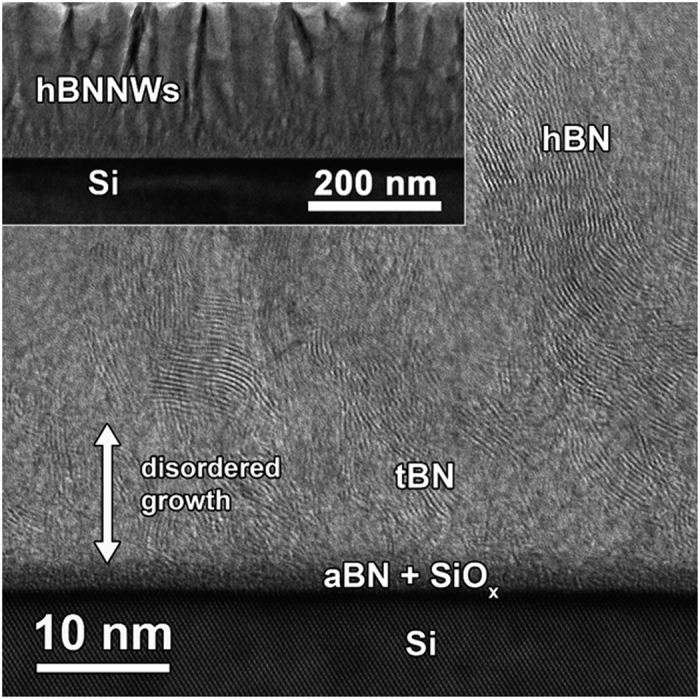
A cross-sectional HRTEM image with bright field cross-sectional TEM image as inset of hBNNWs-Si. These micrographs show the presence of aBN and tBN sequentially prior to the growth of hBN phase when the hBNNWs were grown directly on Si substrates.

**Figure 5:**
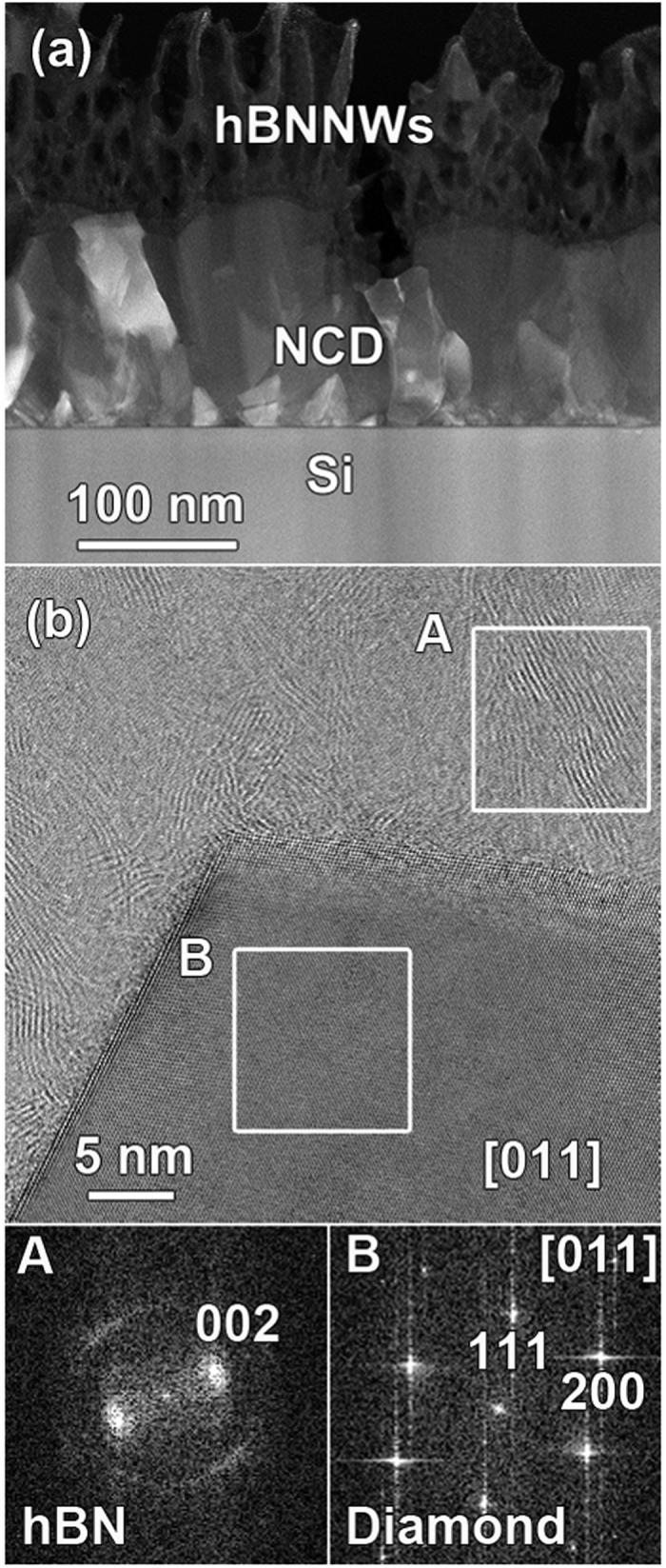
(**a**) Cross-sectional ADF-STEM image of the hBNNWs-NCD heterostructure. (**b**) High resolution ADF-STEM image of the NCD/hBN interface. The FT pattern from region A evidences the crystalline nature of the hBN, displaying the (002) reflection. The diamond particle is imaged along the [011] zone axis, as evidenced by the FT pattern taken from region B.

The microstructure of these heterostructures was examined by annular dark field scanning transmission electron microscopy (ADF-STEM) and high resolution TEM in order to clarify the basis of the FEE enhancement and the PI performance of the hBNNWs-diamond heterostructures. The cross-sectional high resolution TEM image of hBNNWs-Si was shown in Fig. 4 with the corresponding bright field cross-sectional TEM shown as inset in this figure. These micrographs illustrate that when hBN was directly grown on Si-substrates, it requires the formation of precursor layers prior to its nucleation. The deposition of hBN on Si first yields an interlayer of amorphous BN (aBN) followed by the formation of turbostratic BN (tBN) with (002) basal planes almost perpendicular to the substrate surface^62^. The edges of the (002) BN basal planes then serve as nucleation sites of hBN. In contrast, Fig. 5a shows a typical cross-sectional ADF-STEM micrograph of the hBNNWs-NCD heterostructure, in which the hBNNWs and the diamond regions are clearly marked. Figure 5b shows a high resolution image obtained from a typical region at the hBN-diamond interface of the hBNNWs-NCD heterostructure. It can be seen that the hBNNWs grow directly on the diamond surfaces, without the formation of any precursor layers like aBN or tBN prior to its nucleation. Highly ordered lattice fringes of hBNNWs can be observed, indicating that the hBNNWs are well crystallized. Fourier transformed diffractograms corresponding to regions A and B are shown in isnets A and B of Fig. 5b, clearly showing that region A contains hBN materials and region B contains diamond. The thickness of the hBNNWs was measured to be around 10 nm (not shown).

**Figure 6:**
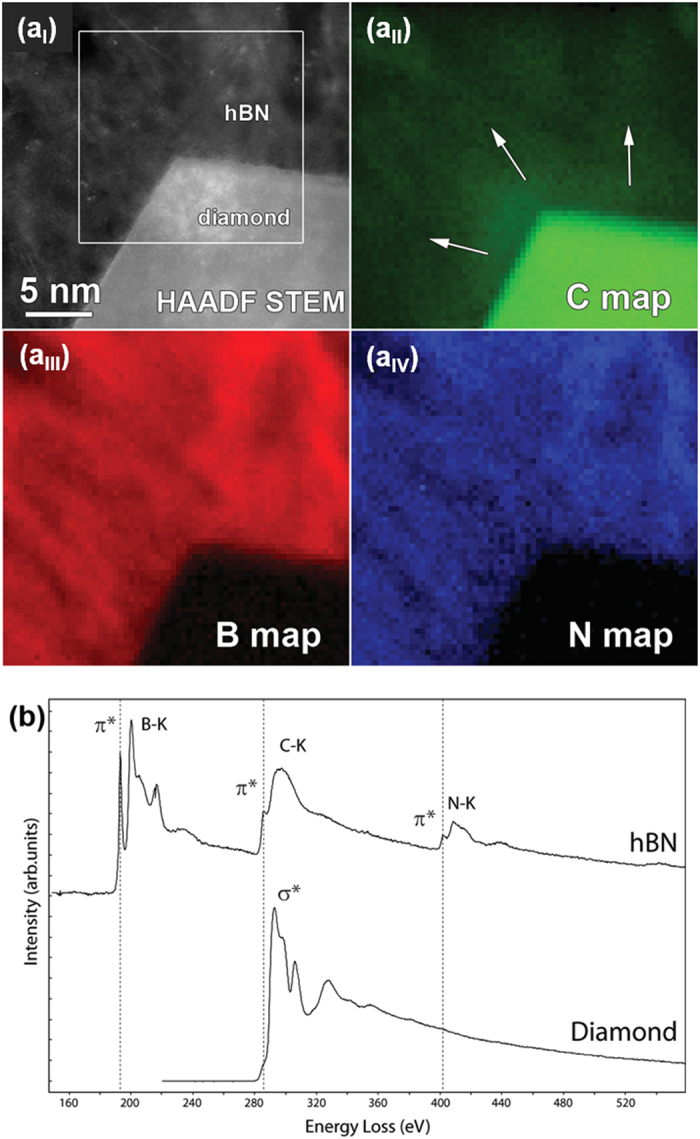
(**a**) ADF-STEM micrograph of the hBNNWs-NCD interface together with EELS elemental maps for carbon, boron and nitrogen taken from the region indicated by the white rectangle (**b**) Summed EELS core-loss spectra taken from the diamond and hBN regions in the maps in (**a**). A significant carbon incorporation into the hBN is evidenced by the carbon map (arrows) and the strong amorphous carbon peak in the EELS spectrum taken from the hBN region.

In order to investigate a possible incorporation of carbon into the hBN, we adopted spatially resolved STEM-electron energy loss spectroscopy (EELS). In the experiment, the region indicated by the rectangle in Fig. 6aI was scanned using a fine probe, collecting a core-loss EELS spectrum containing the C-K, B-K and N-K edges in each point. By integrating the intensity under the C, Band N edges, elemental maps were generated that are displayed in Fig. 6aII,III,IV, respectively. It is clear from the maps that some carbon has been incorporated into the hBN structures (indicated by arrows in Fig. 6aII). In Fig. 6b, two summed EELS spectra from the diamond and the hBN regions (designated in Fig. 6aI) are plotted. The carbon K-edge spectrum acquired from the diamond region is typical of *sp*^3^-carbon, with a strong σ* contribution at 292 eV and deep valley in 302 eV^63^,^64^. The EELS spectrum corresponding to the hBN region of Fig. 6a exhibits two distinct edges; the boron-K 188 eV and the nitrogen-K at 401 eV^65^,^66^,^67^. The fine structure of the B-K and N-K edges are typical of the *sp*^2^-coordinated layered BN, indicating that the obtained nanowalls are BN. As mentioned above, in addition to the core-loss K-edges of B and N, the presence of carbon is also detected through presence of a core-loss carbon-K edge at 284 eV (π* band). The fine structure of the carbon K-edge is typical of sp^2^-bonded carbon, confirming that some carbon is incorporated into the hBN. These EELS results together with the elemental maps (cf. Fig. 6aII–IV) indicate the presence of C, B and N species in the hBNNWs-NCD heterostructures, which is in accord with the B-C, B-N, and C-N bonds observed in the FTIR data (cf. Fig. 2). Notably, Leung *et al*. also observed the diffusion of C in the interface during the growth of cubic BN on amorphous tetrahedral carbon interlayers^68^. The presence of C in the interface region is possibly induced by carbon incorporation and dynamic recoil ion mixing in an early stage of boron nitride deposition. This incorporated carbon region then relates to a C-B-N gradient layer, which may contribute to the interfacial stress relaxation. On the basis of FTIR and STEM-EELS observations, it is obvious that the hBNNWs nucleated and grew directly on the NCD surface, inhibiting the formation of aBN and disordered BN phases in the interface. Presumably, the possible interaction of B-C-N species at hBNNWs-to-NCD interface is the main factor, which facilitates the nucleation of hBN clusters without the formation of intermediate BN phases (aBN or tBN).

As mentioned earlier, for a high-quality electron field emitter, an adequate supply of electrons from the substrate (the Si) to the emitting site (the hBNNWs) is critical, besides the low work function for the emitting surface. In order to enhance the efficiency of the electron supply, the conductivity of both hBN and diamond and the resistance of the hBN-to-Si interface need to be optimized. The utilization of diamond as intermediate layer, which was coated onto the Si substrates prior to the growth of hBNNWs, fulfills these critical requirements simultaneously. As illustrated by the TEM investigations, the use of diamond intermediate layer suppresses the formation of an aBN phase and tBN. The hBN forms directly on diamond that enhances the transport of electrons crossing the hBN-to-diamond interface. Concerning the mechanism that using nNCD films as intermediate layer resulted in better EFE behavior compared with those which used NCD as intermediate layer, the induction of a *sp*^2^ graphite phase in the grain boundaries of the nNCD films (evident from micro-Raman studies, cf. spectrum III, Fig. 1e) due to the addition of N_2_ in the plasma^69^ is presumed to be the main factor. As the *sp*^2^ graphitic phase increased the percolative conduction paths in the nNCD films that could be one of the factors for the improvement in FEE properties for hBNNWs-nNCD heterostructures, as compared to hBNNWs-NCD. Meanwhile, it is important to notice that the graphite phase in the grain boundaries of nNCD films is intrinsically not resistant to plasma ion bombardment^70^ that lead to inferior robustness of nNCD-based microplasma devices compared with that of NCD-based ones.

Now, an unresolved question rises on how the unique combination of hBNNWs and nNCD materials assists in enhancing the FEE properties and what would be the possible field emission mechanism in hBNNWs-nNCD heterostructures? Basically hBN is of a structure analogous to graphite but exhibits a large bandgap of 5.95 eV^71^, viz. hBN is an insulating layered material. To make a specific application of hBN in nanoelectronics, it is important to modify the electronic properties of hBN by doping it with suitable dopant elements. Particularly, when doped carbon, hBN exhibited semiconducting properties due to the appearance of dopants or defect states in the bandgap^72^,^73^. In the present study, it has been observed from FTIR (cf. Fig. 2) and STEM-EELS (cf. Fig. 6) that there is incorporation of carbon in the hBNNWs, resulting in the formation of C-N and B-C bonding in hBN region which could improve the electrical conductivity of hBNNWs. From these observations, the field emission mechanism for the improvement in the FEE behavior of hBNNWs-nNCD heterostructures can be enlightened: first, the *sp*^2^ graphite phase in the grain boundaries of nNCD films conducts the electrons efficiently to the hBNNWs-nNCD interface. Second, the direct growth of hBN on the diamond surface lowers the resistivity of the interfacial layer and therefore the electrons can be transferred readily from nNCD films across the interfacial layer to the hBNNWs. Finally, the incorporation of C in the hBNNWs provides efficient electron transport paths for the emitted electrons to reach the tip of the nanowalls from which they escape into vacuum without any difficulty as the hBN surfaces are NEA in nature^9^,^10^ All these factors reduce the *E*_0_ value by lowering the barrier for the emitting electrons and thus enhances the FEE *J*_e_. Moreover, the vertically aligned nanowall structure facing the anode could be considered as an additional reason for improvement of the FEE properties of hBNNWs-nNCD heterostructures.

## Conclusions

A facile and reproducible way of fabricating vertically aligned hBNNWs-nNCD heterostructures with excellent FEE and PI performances is demonstrated. The hBNNWs grown directly on the nNCD surfaces markedly enhance the FEE properties (viz. low *E*_0_ (15.2 V/μm), high *J*_e_ of 1.48 mA/cm^2^ at an applied field of 21.3 V/μm and high life-time stability of 248 min). Such an improvement in the field emission behavior originates from the unique material combination used. The cross-sectional TEM results show that, in the hBNNWs-diamond heterostructures, hBN nucleated and grew directly on the diamond surfaces, eliminating the formation of an aBN/tBN phase. There is also incorporation of C in the hBN region, which leads to improvement of the conductivity for the hBNNWs. Moreover, the addition of N_2_ in the diamond growth plasma induces *sp*^2^-bonded carbon phases in the grain boundaries of the NCD films that improve the electron transport from diamond to the hBNNWs, resulting in the enhanced FEE properties of hBNNWs-nNCD heterostructures. The potential application of these heterostructures is demonstrated by the PI measurements for a microplasma device, that is, the lowering of the threshold voltage by 370 V and the increase in plasma current density to *J*_pl_ = 2.48 mA/cm^2^. These observations confirmed the role of the diamond interlayer in the enhancement of the electron emission behavior of hBNNWs-nNCD heterostructures. Consequently, the present approach for fabricating hBNNWs-nNCD heterostructures is a simple and a direct process that opens new aspects in flat panel displays and high brightness electron sources.

## Methods

The Si substrates were kept in an ASTeX 6500 series MPECVD reactor for the growth of diamond films. Prior to diamond growth, the Si substrates were seeded with a waterbased state-of-the-art colloidal suspension of 5 nm detonation nanodiamonds^74^. In the growth of undoped NCD films, a gas mixture of CH_4_ and H_2_ with flow rates of 3 and 297 sccm (CH_4_/H_2_ = 1/99), respectively, was excited by 3000 W microwave power. The total pressure in the chamber was maintained at 20 Torr. The substrates were heated due to the bombardment of the plasma species and the growth temperature was estimated to be around 675 °C during the growth of undoped NCD films. The growth temperature was measured using an optical pyrometer. For the growth of nNCD films, a gas mixture of CH_4_, H_2_ and N_2_ with flow rates of 18, 267 and 15 sccm, respectively (CH_4_/H_2_/N_2_ = 6/89/5), was excited by 3000 W microwave power, and the total pressure in the chamber was maintained at 20 Torr. The growth temperature during the growth of nNCD films was estimated to be around 540 °C.

hBNNWs were synthesized on the NCD films by a home-built unbalanced 13.56 MHz RF sputtering technique. An optimal condition of fabrication was used with a gas mixture Ar(51%)/N_2_(44%)/H_2_(5%) and cathode power of 75 W. Herein, a 3 inch-diameter pyrolytic BN ceramic target (Kurt J. Lesker) was used with material purity and mass-density of 99.99% and 1.96 × 10^3^ kg/m^3^, respectively. The working pressure and target-to-substrate distance were 2.1 × 10^−2^ mbar and 3 cm, respectively. The growth temperature during growth of hBNNWs was 125 °C^31^.

The samples were characterized by micro-Raman spectroscopy, FTIR spectroscopy, SEM, HRTEM, ADF-STEM and STEM-EELS using, respectively, a Horiba Jobin-Yuan T64000 spectrometer, FTIR NICOLET 8700 spectrometer, and FEI Quanta 200 FEG microscope, a JEOL 3000F transmission electron microscope operated at 300 kV acceleration voltage for HRTEM and a FEI Titan ‘cubed’ microscope operated at 300 kV for ADF-STEM-EELS. The convergence semi-angle *α* used was 22 mrad, the inner acceptance semi-angle *β* for ADF-STEM imaging was 22 mrad, the EELS collection angle used was also 22 mrad. The TEM specimens of these samples were prepared by Focused Ion Beam technique.

The FEE characteristics of these samples were measured with a tunable parallel plate setup, in which a micrometer was used to control the cathode-to-anode distance. An electrometer (Keithley 237) was used to measure the current-voltage (*I*-*V*) characteristics at pressures below 10^−6^ Torr. The point of intersection of the straight lines extrapolated from the low and high-field segments of the F-N plots, viz. log (*J*_e_/*E*^2^) versus 1/*E*, is labeled as the *E*_0_. The fabrication of a plasma microcavity using hBNNWs-NCD heterostructures as cathodes are given elsewhere^22^.

## Additional Information

**How to cite this article**: Sankaran, K. J. *et al*. Enhanced optoelectronic performances of vertically aligned hexagonal boron nitride nanowalls-nanocrystalline diamond heterostructures. *Sci. Rep.* **6**, 29444; doi: 10.1038/srep29444 (2016).

## Supplementary Material

Supplementary Information

## Figures and Tables

**Table 1:** Comparison of the key field electron emission performance parameters between the presented hBNNWs-nNCD heterostructures and other field emitters.

Materials	Turn-on field (V/μm)	Field enhancement factor	Life-time stability (min)
Carbon nanotubes^52^	1.4	4350	33
Boron nitride nanotubes^53^	32.5	98	—
Carbon nanowalls^54^	4.7	1399	240
AlN nanocones^55^	4.8	1561	—
ZnO Nanowires^56^	3.82	2303	—
MoO_3_ nanobelts^57^	8.7	—	120
Ultrananocrystalline diamond nanorods^58^	2.04	1945	—
Graphene nanoflakes^59^	1.05	3120	166
hBNNWs-nNCD heterostructures^This study^	15.2	3057	248
