# Supplementary material for: Enhanced optoelectronic performances of vertically aligned hexagonal boron nitride nanowalls-nanocrystalline diamond heterostructures
Source: Sci Rep. 2016 Jul 11;6:29444. doi: 10.1038/srep29444 (PMC4941520; doi:10.1038/srep29444)
Supplement: Supplementary Information [file srep29444-s1.pdf]

Supporting Information

Enhanced optoelectronic performances of vertically aligned hexagonal boron nitride nanowalls-nanocrystalline diamond heterostructures

Kamatchi Jothiramalingam Sankaran[1,2], Hoang Duc Quang[1,2], Srinivasu Kunuku[3], Svetlana Korneychuk[4], Stuart Turner[4], Paulius Pobedinskas[1,2], Sien Drijkoningen[1,2], Marlies K. Van Bael[1,2], Jan D'Haen[1,2], Johan Verbeeck[4], Keh-Chyang Leou[3], I-Nan Lin[5], & Ken Haenen[1,2]

[1]Institute for Materials Research (IMO), Hasselt University, 3590 Diepenbeek, Belgium. [2]IMOMEC, IMEC vzw, 3590 Diepenbeek, Belgium. [3]Department of Engineering and System Science, National Tsing Hua University, 30013 Hsinchu, Taiwan. [4]Electron Microscopy for Materials Science (EMAT), University of Antwerp, 2020 Antwerp, Belgium. [5]Department of Physics, Tamkang University, 251 Tamsui, Taiwan. Correspondence and requests for materials should be addressed to K.J.S. and K.H. (email: sankaran.kamatchi@uhasselt.be, ken.haenen@uhasselt.be).

Table S1. Field electron emission and plasma illumination properties of hBNNWs grown on Si and NCD films.

Samples	Field electron emission (FEE)				Plasma illumination (PI)		
	E_0 (V/μm)	J_e (mA/cm^2)	β	τ_e (min)	V_b (V)	J_{pl} (mA/cm^2) @ 500 V	τ_{pl} (min) @ 500 V
hBNNWs-Si	46.6	0.21 @ 91.6 V/μm	560	27 @ 90.0 V/μm	460	0.57	28
hBNNWs-NCD	35.5	0.46 @ 61.3 V/μm	2110	284 @ 53.5 V/μm	430	1.55	163
hBNNWs-nNCD	15.2	1.48 @ 21.3 V/μm	3057	248 @ 19.6 V/μm	370	2.48	122

E_0: the turn-on field for FEE process that was designated as the interception of the lines extrapolated from the high-field and low-field segments of the F-N plots.

J_e: the FEE current density evaluated at the applied field designated.

β: the field enhancement factor.

τ_e: the life-time stability tested under the applied field designated.

V_b: the breakdown voltage for PI process.

J_{pl}: the PI current density evaluated at the applied voltage of 500 V.

τ_{pl}: the life-time stability tested under the applied voltage of 500 V.

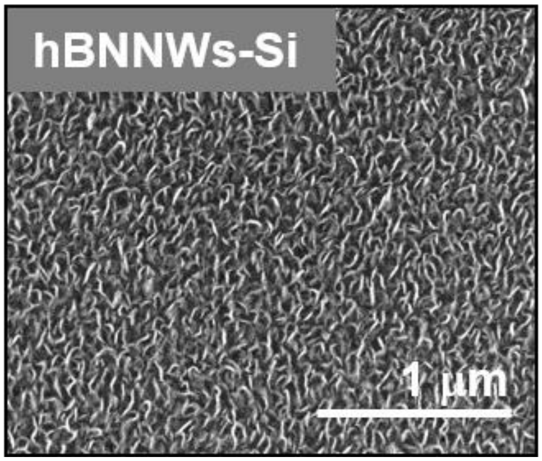

Figure S1. The plane view SEM micrograph of hBNNWs grown on Si. A non-uniform and unbranched growth of hBNNWs was observed.

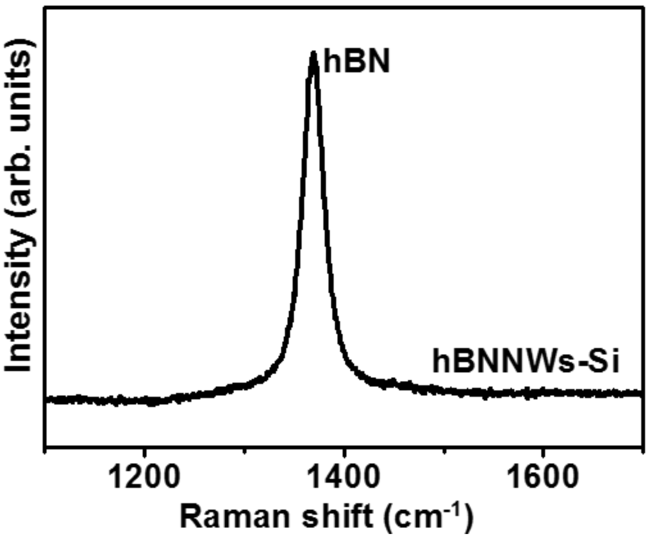

Figure S2. Micro-Raman spectrum from hBNNWs grown on Siwhich contain a major peak that appears at 1368 cm^{-1}, attributed to the high frequency intralayer E_{2g} vibration mode of hBNmaterials.

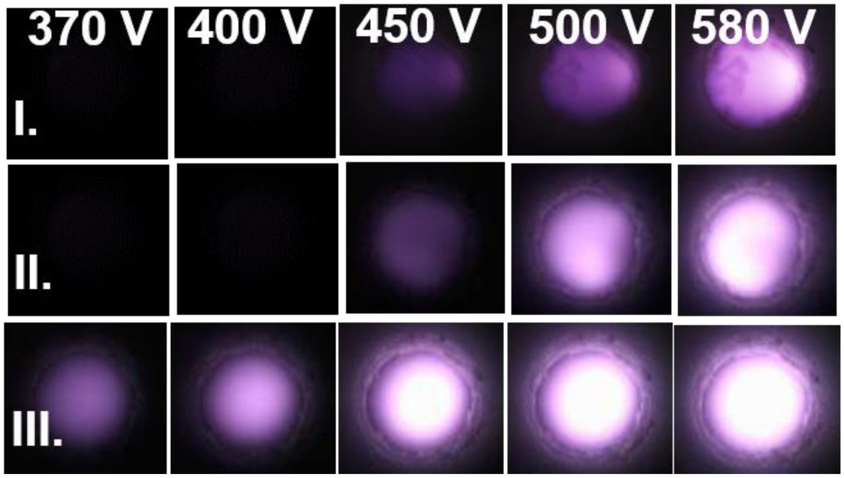

Figure S3. The plasma illumination images of the microplasma cavities, which were fabricated using I. hBNNWs-Si, II. hBNNWs-NCD and III. hBNNWs-nNCD as cathode materials. The microplasma devices using the hBNNWs-nNCD as cathode can be triggered by a voltage as low as 370 V (image series III), whereas those using hBNNWs-NCD as cathode need 430 V to ignite the plasma (image series II). In contrast, the hBNNWs-Si based microplasma devices need a higher voltage, around 460 V (image series I), to trigger the plasma. The plasma intensity enhances monotonically with the applied voltage.
